# Spatial regulation of cytoplasmic snRNP assembly at the cellular level

**DOI:** 10.1093/jxb/erv399

**Published:** 2015-08-27

**Authors:** Malwina Hyjek, Natalia Wojciechowska, Magda Rudzka, Agnieszka Kołowerzo-Lubnau, Dariusz Jan Smoliński

**Affiliations:** ^1^Department of Cell Biology, Faculty of Biology and Environmental Protection, Nicolaus Copernicus University, Lwowska 1, Toruń, 87–100, Poland; ^2^Centre For Modern Interdisciplinary Technologies, Nicolaus Copernicus University, Wileńska 4, 87–100 ToruńPoland; ^3^Department of General Botany, Institute of Experimental Biology, Faculty of Biology, A. Mickiewicz University, Umultowska 89, 61–614 Poznań, Poland

**Keywords:** Cajal bodies, confocal microscopy, in situ hybridization, Larix, P bodies, plant cell, Sm proteins, snRNA, splicing.

## Abstract

Small nuclear ribonucleoproteins (snRNPs) play a crucial role in pre-mRNA splicing in all eukaryotic cells. In contrast to the relatively broad knowledge on snRNP assembly within the nucleus, the spatial organization of the cytoplasmic stages of their maturation remains poorly understood. Nevertheless, sparse research indicates that, similar to the nuclear steps, the crucial processes of cytoplasmic snRNP assembly may also be strictly spatially regulated. In European larch microsporocytes, it was determined that the cytoplasmic assembly of snRNPs within a cell might occur in two distinct spatial manners, which depend on the rate of *de novo* snRNP formation in relation to the steady state of these particles within the nucleus. During periods of moderate expression of splicing elements, the cytoplasmic assembly of snRNPs occurred diffusely throughout the cytoplasm. Increased expression of both Sm proteins and U snRNA triggered the accumulation of these particles within distinct, non-membranous RNP-rich granules, which are referred to as snRNP-rich cytoplasmic bodies.

## Highlight

Under physiological conditions, it was shown that the same cell model may establish two distinct spatial manners of cytoplasmic snRNP assembly.

## Introduction

Controlled gene expression within a cell is fundamental for the functioning of all living organisms. The expression of eukaryotic protein-coding genes is a complex process in which three main steps can be distinguished, namely transcription, pre-mRNA splicing, and translation. The main function of splicing, which is the removal of non-coding intronic sequences from nascent pre-mRNA, requires the function of specialized ribonucleoprotein machinery referred to as a spliceosome. This macromolecular complex consists of uridine-rich small nuclear ribonucleoproteins (U snRNPs) and approximately 200 proteins (Jurica and Moore, 2003; Matera and Wang, 2014). There are five main types of U snRNPs, U1, U2, U4, U5, and U6, which are each composed of a single uridine-rich RNA transcript (U snRNA), a common core domain, and a set of specific proteins (Will and Lührmann, 2001*a*). The common core domain comprises seven highly conserved Sm proteins that create a heteroheptameric ring around the ‘Sm site’ on the U snRNA transcript (Branlant *et al.*, 1982; Baserga and Steitz, 1993; Nagai and Mattaj, 1994; Raker *et al.*, 1999; Kambach *et al.*, 1999; Scofield and Lynch, 2008). The group of eight spliceosomal Sm proteins is referred to as the canonical Sm, and they are encoded by seven genes: B/B’, D1, D2, D3, E, F and G, in which the B and B’ proteins are alternative splicing variants of the same gene (Chu and Elkon, 1991). Although no known RNA-binding region has been identified in the sequence of any particular Sm proteins, it has been proposed that this type of RNA recognition domain is intermolecular and may form as a result of Sm core assembly (Hermann *et al.*, 1995; Raker *et al.*, 1996). Canonical Sm proteins are present in all spliceosomal snRNPs, with the exception of U6 snRNP.

U snRNPs represent an excellent model for studies on ribonucleoprotein biogenesis and the spatial regulation of expression within a cell. Their assembly and maturation is a complex, stepwise process that occurs in both the nucleus and the cytoplasm. All spliceosomal pre-snRNAs, with the exception of U6, are transcribed in the nucleus by RNA polymerase II and acquire an m7G cap. In the next step, transcripts are rapidly exported to the cytoplasm (Askjaer *et al.*, 1999; Segref *et al.*, 2001; Fischer *et al.*, 2011), where each U snRNA binds seven Sm proteins, which thus creates an snRNP core particle (Sauterer *et al.*, 1988; Zieve *et al.*, 1988). It has been shown that, despite the direct assembly of the Sm core on U snRNA *in vitro*, this process *in vivo* is mediated and strictly coordinated with the participation of a large protein complex, referred to as the survival of motor neuron (SMN) (Pellizzoni *et al.*, 2002; Yong *et al.*, 2004; Li *et al.*, 2014). SMN remains associated with snRNPs throughout the cytoplasmic phase of their maturation and guides proper snRNP synthesis (Massenet *et al.*, 2002; Seng *et al.*, 2015). The Sm ring assembly is a multi-step process. Studies on HeLa cells have demonstrated that, prior to assembly on U snRNA, Sm proteins form RNA-free hetero-oligomers (Raker *et al.*, 1996; Kambach *et al.*, 1999). Further investigation has shown that, immediately after translation, Sm proteins are bound by the protein complex PICln (chloride conductance regulatory protein), which serves as an additional control point in the coordination of snRNP synthesis dynamics (Pu *et al.*, 1999; Pellizzoni *et al.*, 2002; Yong *et al.*, 2004). Furthermore, prior to interaction with SMN, inactive Sm proteins are bound to the 20S methylosome, which is responsible for symmetric arginine dimethylation of specific RG (arginine- and glycine-rich) domains in SmB, D1, and D3 (Friesen *et al.*, 2001; Meister *et al.*, 2001). Despite a rich knowledge regarding Sm core assembly with snRNA at the molecular level, the spatial organization of this process in the cytoplasm remains unknown. After Sm core assembly on U snRNA, this domain acts as a binding site for trimethylguanosine synthase, an enzyme that catalyses the hypermethylation of the m7G cap at the 5′ end of the transcript, which results in the creation of a characteristic 2,2,7-m3G cap (Mattaj, 1986; Fischer *et al.*, 2011). During the cytoplasmic maturation of U snRNPs, the 3′ end processing of U snRNA also occurs via exonucleolytic cleavage; however, the details of the nature of this process remain unknown (van Hoof *et al.*, 2000). Hypermethylated snRNP particles are subsequently reimported to the nucleus. This reimport is subject to a bipartite nuclear localization signal concreated by both the Sm core and m3G cap (Fischer *et al.*, 1993; Plessel *et al.*, 1994; Hamm *et al.*, 1990).

The spatial localization of snRNPs after reimport to the nucleus has been well established and encompasses a diffused localization in the nucleoplasm, as well as concentration of these particles in Cajal bodies (CBs), speckles, and nucleoli (Sleeman and Lamond, 1999). Many studies have demonstrated that nuclear maturation of snRNPs is a spatially organized, stepwise process, which includes a chain of sequential processing and maturation that occurs in specialized, spatially distinct nuclear domains. It has been demonstrated that, after nuclear reimport, U snRNPs are first accumulated within CBs (Sleeman and Lamond, 1999), where they undergo the final steps of maturation, i.e. methylation and pseudo-uridylation. These processes are guided by scaRNAs (small Cajal body-specific RNAs), which localize specifically in CBs (Massanet *et al.*, 1998; Jády and Kiss, 2001; Darzacq *et al.*, 2002; Kiss, 2002; Jády *et al.*, 2003). These domains are also sites of functional spliceosomal subunit assembly, including the formation of the U4/U5*U6 snRNP tri-complex (Stanek *et al.*, 2008; Novotný *et al.*, 2011). The process of U snRNP maturation is highly dynamic. It has been demonstrated that, after cytoplasmic assembly, newly formed U snRNPs are rapidly reimported to the nucleus (Sauterer *et al.*, 1988), where they transiently accumulate within CBs and are readily sequestered in interchromatin clusters referred to as speckles (Sleeman and Lamond, 1999).

In contrast to the relatively broad knowledge regarding snRNP assembly within the nucleus, the spatial organization of the cytoplasmic stages of their maturation remains poorly understood. Nevertheless, sparse research indicates that, similar to the nuclear steps, the crucial processes of cytoplasmic snRNP assembly might also be strictly spatially regulated. It has been shown that, in both animal (Liu and Gall, 2007) and plant (Smoliński *et al.*, 2011) cells, Sm proteins, U snRNA, and m3G-capped snRNA accumulate in numerous distinct cytoplasmic foci, which probably represent the sites of snRNP assembly or storage prior to returning to the nucleus.

Here, it was show that, under physiological conditions, the same cell model might establish two distinct spatial manners of cytoplasmic snRNP assembly. Depending on the rate of *de novo* snRNP formation in relation to the steady state of these particles within the nucleus, the cytoplasmic pool of snRNP components is distributed in a dispersed or accumulated manner. During stages of high expression of splicing elements, the cytoplasmic assembly of snRNPs is coordinated by distinct, non-membranous RNP-rich bodies, which probably facilitate the rate of assembly during high levels of certain RNAs and proteins. It is propose here that these cytoplasmic bodies function as a regulatory platform that coordinates the kinetics of *de novo* snRNP formation during the intensive expression of splicing elements, and that they regulate the proper pool of accessible Sm proteins relative to the U snRNA level in the cytoplasm.

## Materials and methods

### Plant material and isolation of meiotic protoplasts

Anthers of European larch (*Larix decidua* Mill.) were collected from the same tree in successive meiotic prophase stages of the diplotene, from November to March at weekly intervals, to ensure constant experimental conditions. Anthers were fixed in 4% paraformaldehyde in phosphate-buffered saline (PBS), pH 7.2, for 12h and squashed to obtain free meiocytes. Meiotic protoplasts were isolated from these cells according to the method of Kołowerzo *et al.* (2009); they were subsequently subjected to double-labelling assays using immunedetection of Sm proteins and fluorescent *in situ* hybridization (FISH) of snRNA with a tyramide signal amplification (TSA) technique.

### Design of double-labelling reactions

Several double-labelling FISH/immunofluorescence assays (U snRNA and Sm proteins) were performed as described below. In the reactions, the *in situ* hybridization method always preceded the immunocytochemical methods. Prior to the assay, the cells were treated with PBS containing 0.1% Triton X-100 for cell membrane permeabilization. After the double-labelling assay, the slides were stained for DNA detection with DAPI and mounted in ProLong Gold antifade reagent (Life Technologies, USA).

### FISH detection of snRNA with the TSA technique

For localization of snRNA, a TSA technique was applied because its detection sensitivity is more than 1000 times higher than standard methods. For hybridization, the probe was resuspended in hybridization buffer [30% (v/v) formamide, 4× SSC, 5× Denhardt’s buffer, 1mM EDTA, and 50mM phosphate buffer] at a concentration of 50 pmol ml^–1^. Hybridization was performed overnight at 26 °C. The following antisense DNA oligonucleotides were used for the reactions, labelled with digoxygenin (DIG): for the detection of U4 snRNA: 5′-DIG-GGAAATAGTTTTCAACCAGCAATAGAC-3′ (Metabion, Germany) and for the detection of U5 snRNA: 5′-DIG-TATTCTTTAGTAAAAGGCGAAAGAATAGTT-3′ (Metabion, Germany). After washing, non-specific antigens were blocked with PBS containing 0.1% acetylated BSA for 30min in a humidified chamber. Next, the probes were detected using primary rabbit anti-DIG antibody (diluted 1:100; Life Technologies) in 0.05% acetylated BSA in PBS in a humidified chamber overnight at 11 °C. The slides were subsequently washed with PBS and incubated for 30min in PBS containing 0.1% acetylated BSA for the blocking of non-specific antigens. Next, the material was incubated with horseradish peroxidase-labelled goat anti-rabbit secondary antibody (diluted 1:1000; Life Technologies) in 0.05% acetylated BSA in PBS in a humidified chamber for 1h at 36 °C. The reaction was visualized with the use of tyramide, which was conjugated with Alexa Fluor 488 in 0.0015% H_2_O_2_ (diluted 1:200; Life Technologies), and incubated at room temperature in a humidified chamber for 10min (according to MP 20911 protocol, Life Technologies).

### Immunodetection of Sm proteins

Primary mouse Y12 antibody (a gift from Karla Neugebauer, Max Planck Institute of Molecular Cell Biology and Genetics, Dresden, Germany) was used for the detection of Sm proteins, diluted 1:20 in PBS containing 0.01% acetylated BSA, and incubated at 8 °C in a humidified chamber overnight. After washing with PBS, the slides were incubated with secondary goat anti-mouse antibody conjugated with Alexa Fluor 546 (Life Technologies), diluted 1:100 in PBS containing 0.01% acetylated BSA, and incubated at 36 °C in a humidified chamber for 1h.

### Microscopic and quantitative measurements

The results were registered with a Nikon PCM 2000 confocal microscope using an argon/ion laser that emitted light with a wavelength of 488nm (blue excitation and green fluorescence) and a He/neon laser that emitted light with a wavelength of 543nm (green excitation and red fluorescence). A mid-pinhole, long exposure time (75 µs), and a 100× (numerical aperture, 1.4) Plan Apochromat DIC H oil immersion lens were used. Pairs of images were collected simultaneously in the green (Alexa Fluor 488 fluorescence) and red (Alexa Fluor 546 fluorescence) channels. For DAPI staining, an inverted Nikon Eclipse TE 2000 fluorescence microscope equipped with a mercury lamp, a UV-2EC UV narrow-band filter, and a DXM 1200 FX digital camera was used. For quantitative measurements, each experiment was performed using consistent temperatures, incubation times, and concentrations of probes and antibodies. Eight to 20 cells from each stage were analysed, depending on the stage. Three-dimensional optical sections were acquired with a 0.5 µm step interval. For all antigens and developmental stages, the obtained data were corrected for background autofluorescence, as determined by negative-control signal intensities. For the image processing and analysis, the EZ Viewer software package (Nikon Europe BV, Badhoevedorp, The Netherlands) was used. For signal evaluation and colocalization analysis, CeSa Statistical Analyser (Department of Cell Biology, Nicolaus Copernicus University, Toruń, Poland) software was used. The signal intensity µm^–3^ was expressed in arbitrary units of fluorescence intensity. Statistical analysis was performed using PAST software (Hammer *et al.*, 2001). To compare all groups and to determine if there were significant group differences, a non-parametric Kruskal–Wallis test was used. To identify specific group differences, a Mann–Whitney *U* test with Bonferroni correction was used. Correlation analysis was performed with the use of Pearson’s correlation coefficient.

### Control reactions

For the immunofluorescence methods, control incubations lacking the primary antibody were performed. For the *in situ* hybridization with the TSA method, control reactions, which included omission of the probe, primary, or secondary antibody, were performed. All control reactions produced negative results, or the result of the control reaction was undetectably low compared with the standard reactions.

## Results

### Larch microsporocytes exhibit two distinct spatial patterns of snRNP assembly

**Fig. 1.**
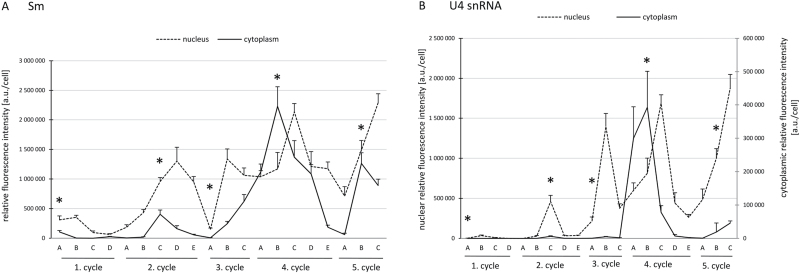
Quantitative analysis of Sm protein (A) and U4 snRNA (B) cytoplasmic and nuclear levels during diplotene in larch microsporocytes. Relative fluorescence intensity is given as arbitrary units (a.u.) per cell. Five similar cycles of the amount and distribution of the molecules are highlighted. Each cycle consisted of three to five stages, named A–E. Asterisks represent stages of cytoplasmic snRNP assembly during each cycle. Results are shown as means±SE.

**Fig. 2.**
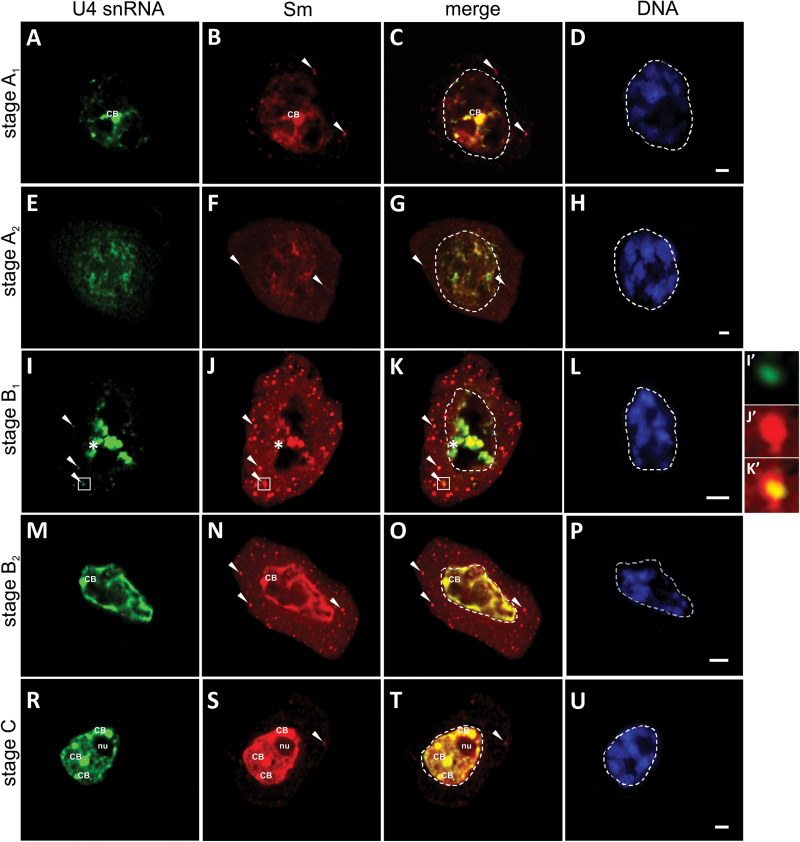
Double labelling of U4 snRNA and Sm proteins during the fourth cycle of synthesis. Stages A and B are divided in two substages referred to as A_1_ and A_2_ for stage A and B_1_ and B_2_ for stage B because of the distinct snRNP patterns of localization. (A–D) Stage A_1_. Nuclear U4 snRNA fluorescence was visible (A), most of which colocalized with the Sm signal (B, C). Additionally, spherical 2 µm diameter foci of Sm signal accumulation were observed in the cytoplasm (B, arrowheads), lacking U4 snRNA (C, arrowheads). (E–H) Stage A_2_. The U4 snRNA signal localized to the entire nucleus, with noticeable irregular accumulations of transcripts (E) devoid of the Sm signal (F, G). In the cytoplasm, distinguishable foci of Sm accumulation remained visible (F, G, arrowheads). (I–L) Stage B_1_. The U4 snRNA staining indicated a significant enrichment of transcripts at the border between the nucleus and cytoplasm (I, asterisk), lacking corresponding Sm accumulation (J, K, asterisk). Within the cytoplasm, the U4 snRNA was localized in large cytoplasmic clusters (I, arrowheads, inset I’), which colocalized with Sm proteins (J, K, arrowheads, insets J’, K’). (M–P) Stage B_2_. Strong nuclear U4 snRNA and Sm staining showed a dispersed pattern of localization within the nucleoplasm, with distinct accumulation in individual CBs that formed in close proximity to the nuclear envelope (M–O). No cytoplasmic clusters of U4 snRNA fluorescence were visible (M), whereas Sm staining still exhibited numerous Sm-rich cytoplasmic granules (N, O, arrowheads). (R–U) Stage C. The nuclear signal of U4 snRNA and Sm fluorescence showed a more evenly distributed pattern within the nucleoplasm (R, S); the CBs were located throughout the whole nucleus and were frequently associated with the nucleolus (R–T). In the cytoplasm, there were Sm-containing clusters that remained visible (S, T, arrowheads), which lacked U4 snRNA (R). The corresponding DAPI images were collected using wide-field fluorescence (D, H, L, P, U). nu, Nucleolus. Bars, 10 µm.

**Fig. 3.**
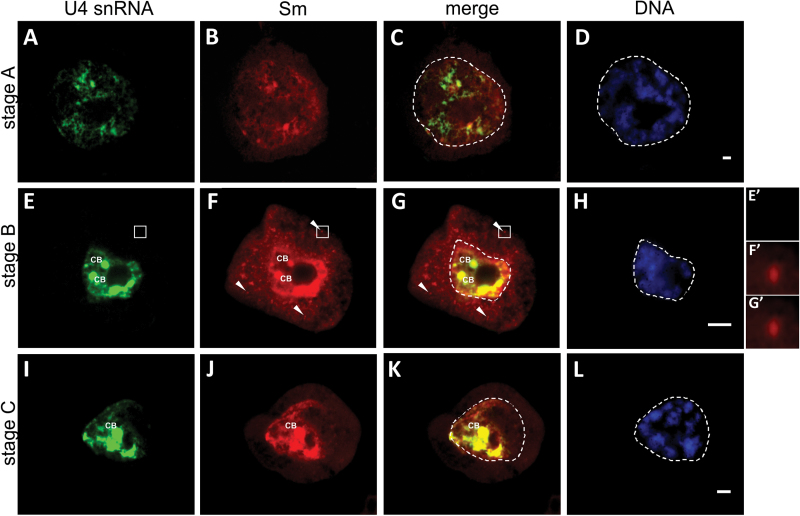
Double labelling of U4 snRNA and Sm proteins during the fifth cycle of synthesis. (A–D) Stage A. Nuclear U4 snRNA staining was visible (A), with a noticeable portion of nucleoplasmic signals devoid of Sm accumulation (B, C). The cytoplasmic signal from Sm localization was dispersed throughout the cytoplasm, and no accumulation of Sm in distinct clusters was present (B). (E–H) Stage B. The U4 snRNA signal showed a diffused pattern of localization within the nucleoplasm (E); it was also enriched in numerous CBs, which colocalized with Sm staining (E–G). The cytoplasmic Sm localization was dispersed, with numerous discrete accumulations (F, G, arrowheads, insets F’, G’), lacking U4 snRNA (E, inset E’). (I–L) Stage C. An increased level of nuclear Sm staining was visible, which colocalized with U4 snRNA in both the nucleoplasm and the CB (I–K). In the cytoplasm, the Sm fluorescence signal indicated only a dispersed pattern (J). The corresponding DAPI images were collected using wide field fluorescence (D, H, L). Bars, 10 µm.

To perform an analysis of snRNP assembly at the cellular level, a double-labelling assay of U4 snRNA and Sm proteins was employed in larch microsporocytes during the first meiotic prophase stage. The diplotene stage was selected for this experiment, based on its extraordinarily long duration in larch (approximately 5 months), which enabled us to investigate the subsequent stages of snRNP biogenesis with the potential to distinguish the nuclear and cytoplasmic events of snRNP assembly. Analysis of the spatial and temporal distributions of Sm proteins and U4 snRNA demonstrated the occurrence of cyclic changes in both the levels and localization patterns of the splicing elements. The occurrence of five cycles of snRNP biosynthesis could be distinguished during the period investigated (Fig. 1), in which the patterns of Sm and snRNA cellular localization reflect the sequence of molecular events that occur during snRNP assembly. Considering the highest level of staining (Fig. 1), the pattern of snRNP distribution was demonstrated in an example of the two last cycles (Figs 2 and 3). Surprisingly, the analyses revealed that the cycles investigated differed in the localization patterns of Sm and snRNA during the cytoplasmic stage of snRNP assembly.

In the first stage of the fourth snRNP synthesis cycle (stage A_1_), the levels of U4 snRNA in the nucleus were relatively high (Figs 1B and 2A). Most signals colocalized with Sm proteins, particularly in the centrally located CB (Fig. 2A–C). However, a noticeable portion of nucleoplasmic U4 snRNA localization was devoid of the Sm signal (Fig. 2C). In the cytoplasm, numerous spherical 2 µm diameter foci of Sm signal accumulation were observed, which lacked U4 snRNA (Fig. 2B, C, arrowheads).

In the next stage (stage A_2_), significant changes in both Sm and U4 snRNA localization were identified. The nuclear staining was dispersed, with noticeable irregular accumulations of U4 snRNA (Fig. 2E, G). No CBs were observed. During this stage, a significant increase in the levels of the investigated molecules was noted in the cytoplasm (Fig. 1). The Sm proteins exhibited a dispersed pattern of localization, with an apparent region of accumulation in distinct cytoplasmic structures (Fig. 2F, G, arrowheads).

The third stage (stage B_1_) was characterized by the highest cytoplasmic levels of both Sm and U4 snRNA during the entire diplotene stage (Fig. 1). The Sm proteins exhibited dispersed patterns of staining and formed numerous spherical cytoplasmic structures with the largest volume during the whole cycle (Fig. 2J compared with 2F). There were significant changes in the U4 snRNA distribution. The transcripts were localized in large cytoplasmic clusters, which colocalized with Sm proteins (Fig. 2I’, K’). These clusters are referred to as snRNP-rich cytoplasmic bodies (CsBs), as described previously by Smoliński *et al.* (2011). No dispersed signal from U4 snRNA was identified in the cytoplasm during this stage. Additionally, the transcripts were enriched within the nucleus at the nuclear–cytoplasmic border of the cell (Fig. 2I, asterisk). However, no significant accumulation of Sm proteins was observed within this area (Fig. 2J, asterisk).

During the fourth stage (stage B_2_), the distribution of the investigated molecules changed significantly. During this stage, the cytoplasm exhibited a noticeable decrease in U4 snRNA levels, and there were no U4 snRNA clusters visible (Fig. 2M). The decrease in the cytoplasmic U4 snRNA levels correlated with a significant increase in these transcript levels in the nucleus. The staining exhibited a dispersed pattern of localization within the nucleoplasm, with distinct accumulation in individual small CBs that formed in close proximity to the nuclear envelope (Fig. 2M). The level and distribution of Sm proteins in the cytoplasm was similar to the previous stage (Fig. 2N). However, the nuclear localization was significantly different and colocalized with U4 snRNA staining (Fig. 2O). The Sm proteins occurred in dispersed nucleoplasmic form accompanied by accumulation in single CBs, which formed at the nuclear border (Fig. 2N).

In the fifth stage (stage C), the cytoplasmic distribution of U4 snRNA was similar to the previous stage. The transcript levels in this compartment decreased continuously with no cytoplasmic foci identified (Fig. 2R). The nuclear signal exhibited a more evenly distributed pattern within the nucleoplasm, and the CBs were located throughout the whole nucleus and were frequently associated with the nucleolus (Fig. 2R). The cytoplasmic distribution of Sm proteins changed during this stage. The staining indicated very low levels of dispersed signal. Similarly, Sm-containing cytoplasmic clusters were still identified; however, there were substantially fewer clusters per cell visible compared with the previous stage (Fig. 2S compared with 2N).

The final stages of the fourth snRNP synthesis cycle (stages D and E) exhibited a similar distribution pattern for both Sm proteins and U4 snRNA (data not shown), with continuously decreasing levels of these molecules in the cell (Fig. 1).

The next snRNP synthesis cycle lasted for a shorter period of time, which made it impossible to observe as many stages of synthesis as the previous cycle. Thus, the pattern of distribution identified during this cycle was slightly different from the previous cycle; however, the general molecular events, including the nuclear and cytoplasmic phases of snRNP synthesis, remained distinguishable. It is also worth noting that this pattern of snRNP distribution was also similar for the three first cycles that occurred during the diplotene stage (Fig. 1 and data not shown).

In the first stage (stage A), similar to the previous cycle, the nuclear level of U4 snRNA was relatively high (Fig. 1B) and partially colocalized with Sm protein staining (Fig. 3A, C). A noticeable portion of the nucleoplasmic signal was devoid of Sm accumulation (Fig. 3C). In contrast, the pattern of cytoplasmic Sm protein staining was different from the previous cycle. Namely, the signal was dispersed throughout the cytoplasm, and no accumulation of Sm proteins in distinct clusters was visible (Fig. 3B).

The second stage (stage B) is equivalent to the third and fourth stages (B1 and B2) of the previous cycle. During this stage, U4 snRNA exhibited a noticeable increase in both the nucleus and the cytoplasm. The nuclear signal was dispersed within the nucleoplasm and was also enriched in numerous CBs, where it colocalized with Sm proteins (Fig. 3E–G). The cytoplasmic staining of the transcript was dispersed; however, due to high nuclear staining, it was not possible to show it on the image (Fig. 3E). Nevertheless, quantitative analysis unambiguously indicated that, during this stage, the cytoplasmic U4 snRNA level was significantly higher than the other stages of the cycle (Fig. 1B). Furthermore, the Sm proteins exhibited increased levels in both the nucleus and the cytoplasm (Fig. 1A). The cytoplasmic signal was dispersed, with numerous distinct accumulations; however, these accumulations were smaller in diameter compared with the previous cycle (Fig. 3F). Furthermore, these foci did not accumulate U4 snRNA (Fig. 3E’–G’).

The last stage of the fifth cycle (stage C) is also the last stage of diplotene. The localization and high levels of U4 snRNA in the nucleus were similar to the second stage. The level of nuclear Sm staining increased noticeably, where it colocalized with U4 snRNA in both the nucleoplasm and the CBs (Fig. 3J, K). In contrast, the cytoplasmic level of Sm proteins decreased, which correlated with the disappearance of clusters and resulted in only a dispersed localization pattern (Fig. 3J).

**Fig. 4.**
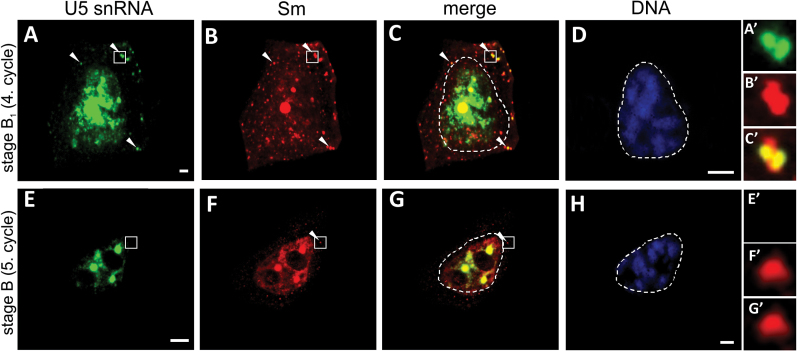
Colocalization of U5 snRNA and Sm proteins during late diplotene. (A–D) Stage B_1_ during the fourth cycle of synthesis. Numerous cytoplasmic foci enriched in both U5 snRNA (A, arrowheads, inset A’) and Sm (B, arrowheads, inset B’) are visible (C, arrowheads, inset C’). (E–H) Stage B during the fifth cycle of synthesis. The cytoplasmic signal from Sm localization was dispersed throughout the cytoplasm with discrete accumulations (F, G, arrowheads, insets F’, G’) that lacked U5 snRNA (E, inset E’). The corresponding DAPI image was collected using wide field fluorescence (D, H). Bars, 10 µm.

To determine whether the two patterns of cytoplasmic localization of snRNA during the diplotene stage are features characteristic for U4 snRNA only or if it is a general snRNA trait, double localization of Sm proteins with U5 snRNA was performed. The staining pattern of U5 snRNPs was parallel to that of U4 snRNPs during the entire period investigated (Fig. 4 and Supplementary Figs S1 and S2, available at *JXB* online). The distribution analysis confirmed that, during the fourth cycle, U5 snRNAs accumulated in CsBs (Figs 4A’–C’ and S1I’–K’), whereas during the first three cycles and the fifth cycle of snRNP synthesis, the cytoplasmic localization of snRNPs occurred in a dispersed manner (Figs 4E’–G’ and S2E’–G’), as shown for U4 snRNA.

### *De novo* formation of Sm-rich cytoplasmic bodies is dependent on the ratio between *de novo*-formed molecules and their total nuclear pool

**Fig. 5.**
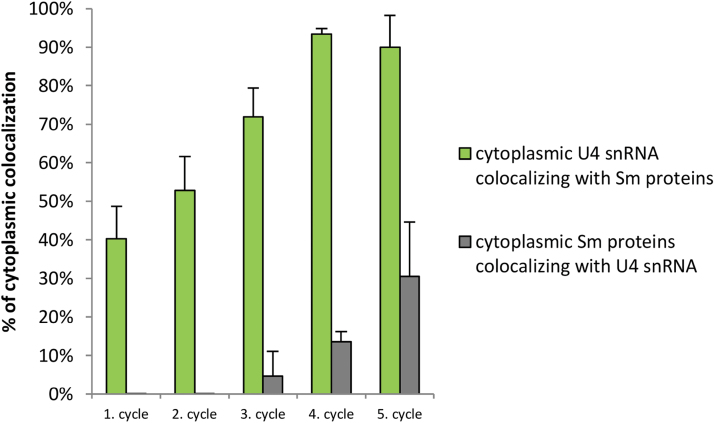
Colocalization analysis of splicing elements during the cytoplasmic stages of snRNP assembly. Columns representing the percentage of U4 snRNA that colocalized with Sm proteins relative to the entire cytoplasmic pool of transcripts, and the percentage of Sm proteins that colocalized with U4 snRNA relative to the entire cytoplasmic pool of the proteins are indicated. Results are shown as means±SE. (This figure is available in colour at *JXB* online.)

To determine whether the occurrence of the five snRNP cycles of biogenesis observed *in situ* correlated with the exchange of these molecules between the nucleus and cytoplasm, a quantitative analysis of Sm proteins and U4 snRNA levels was performed in both compartments (Fig. 1). The measurements indicated that the cyclic changes in both Sm and U4 snRNA distribution identified at the microscopic level corresponded to the sequence of events that occurred during the following stages of snRNP biogenesis. Quantitative analysis also confirmed that the stages of the highest cytoplasmic Sm and U4 snRNA levels during each cycle comprised stages of cytoplasmic snRNP assembly prior to returning to the nucleus. This finding was confirmed by two observations. First, colocalization analysis demonstrated that, during these stages, the percentage of the total cytoplasmic U4 snRNA pool that colocalized with Sm proteins was at a high level (40–96% depending on the cycle, Fig. 5). Secondly, the stages of the highest cytoplasmic Sm and U4 snRNA levels (Fig. 1, asterisks) were followed by a significant decrease in both levels in this compartment, which correlated with a rapid increase in the nucleus (Fig. 1). Pearson’s correlation analysis showed a very high correlation between these events for Sm proteins (*r*=–0.88) and a moderate correlation for U4 snRNA (*r*=–0.48). The lower correlation of cytoplasmic to nuclear levels for U4 snRNA should not be surprising, given that the increase in these molecules in the nucleus may result not only from reimport from the cytoplasm but also from *de novo* transcription of nascent pre-snRNA.

**Fig. 6.**
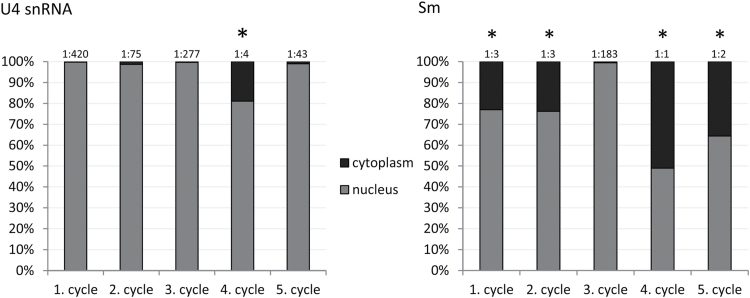
Analysis of the cytoplasmic:nuclear ratio of U4 snRNA and Sm proteins in larch microsporocytes during the stages of cytoplasmic snRNP assembly. Asterisks represent the stages of accumulation of the investigated molecules in distinct cytoplasmic clusters.

Surprisingly, our microscopic observations indicated that, during the diplotene stage, the formation of CsBs occurred exclusively during the fourth cycle of synthesis; Sm proteins colocalized with both U4 and U5 snRNA in distinct spherical clusters (Figs 2I–K and S1I–K). During the other four cycles, U4 and U5 snRNA in cytoplasm was localized only in a dispersed manner (Figs 3E and 4E). Based on these observations, together with a quantitative analysis that demonstrated that, during the fourth cycle, the cytoplasmic U4 snRNA level was the highest of the entire diplotene stage and was several times higher compared with other cycles (Figs 1B and Supplementary Fig. S3), the next aspect to determine was whether a high cytoplasmic level of the molecules of interest was a factor that induced the accumulation of snRNP elements in the cytoplasmic bodies. Thus, an analysis was performed of the cytoplasmic Sm protein levels during the subsequent cycles of snRNP synthesis because Sm-rich cytoplasmic clusters were observed during the first, second, fourth, and fifth cycles. The analysis confirmed that the higher levels of Sm proteins in particular stages were correlated with the occurrence of cytoplasmic bodies (Figs 1A and Supplementary Fig. S3, available at *JXB* online). To analyse further the *de novo* formation of snRNP-rich bodies in relation to the levels of splicing elements within the cell, an analysis was performed of the cytoplasmic:nuclear ratio of Sm and U4 snRNA during specific synthesis cycles (Fig. 6). The analysis demonstrated that the enrichment of these molecules in distinct punctate structures depended on the ratio between the *de novo*-formed molecules (expressed by the cytoplasmic level of the molecule) and the total nuclear pool of these molecules (Fig. 6). This result turned out to be the case for both U4 snRNA and Sm proteins, which is clearly shown in Fig. 6. When the cytoplasmic:nuclear ratio of the molecules investigated was approximately 1:43 or lower, the particles were localized in a dispersed manner throughout the cytoplasm (Figs 3 and 6). A ratio of 1:4 or higher triggered molecule accumulation in distinct spherical cytoplasmic structures (Figs 2 and 6).

## Discussion.

### Spatial organization of snRNP assembly

Based on analysis of the spatial distribution of splicing elements together with quantitative measurements, our research demonstrated the occurrence of five distinct cycles of snRNP biogenesis during diplotene in larch microsporocytes. To the best of our knowledge, this is the first report to demonstrate the spatial organization of snRNP synthesis at the cellular level, with well-marked distinctions between the cytoplasmic and nuclear stages of assembly of splicing elements. The analysis of snRNP biogenesis at the cellular level is considered difficult mainly because of the fact that this process is highly dynamic, especially the cytoplasmic phase (Sauterer *et al.*, 1988; Zieve *et al.*, 1988; Fischer *et al.*, 2011). Thus, the observation of the exchange of snRNAs between the nucleus and cytoplasm in traditional cell models is problematic. European larch microsporocytes represent a suitable cell model for the investigation of spatial organization of snRNP synthesis at the cellular level. Because of the increased metabolic activity, as well as natural fluctuations in the RNA and protein levels of synthesis and distribution (Smoliński *et al.*, 2007; Kołowerzo *et al.*, 2009; Kołowerzo-Lubnau *et al.*, 2015), it was possible to identify and analyse the spatial localization of a multi-step cycle of splicing element biosynthesis within a cell. Nevertheless, due to lack of research regarding snRNP spatial regulation in other plants, particularly at the cytoplasmic stage, it cannot yet be evaluated whether the localization pattern observed in larch will be generally applicable to other species.

It was demonstrated that, during larch meiosis, the cytoplasmic stage of snRNP assembly can occur diffusely throughout the cytoplasm or within spatially distinct microdomains, referred to as CsBs (Smoliński *et al.*, 2011). In light of recent studies, many cytoplasmic post-transcriptional RNA processing steps occur in highly specialized microdomains, which are referred to as cytoplasmic bodies (Moser and Fritzler, 2010; Lavut and Raveh, 2012). In animal cells, the only structures involved in cytoplasmic snRNP assembly and maturation that have been described to date are U bodies. Liu and Gall (2007) showed that these microdomains are probably the sites of Sm core association and U snRNP assembly prior to their reimport to the nucleus. These distinct cytoplasmic snRNP-rich foci have been identified in several species, including human (HeLa) and amphibian (*Xenopus laevis*) cells, as well as in a variety of *Drosophila* tissues (Liu and Gall, 2007; Lee *et al.*, 2009; Buckingham and Liu, 2011). The occurrence of CsBs has also been described previously in larch microsporocytes during pre-meiotic and early meiotic stages (Smoliński *et al.*, 2011). It has been demonstrated that distinct cytoplasmic foci enriched in Sm proteins, U1 snRNA, U2 snRNA, and m3G-capped snRNA occur periodically during the cycles of snRNP synthesis in these cells.

Quantitative analysis indicated that a high level of snRNA and Sm protein expression is probably the inducing factor for CsB formation. This finding is consistent with our previous studies, which showed that, during the early stages of meiosis, CsBs occur in the phases of increased Sm protein expression (Smoliński *et al.*, 2011). It has been well established that *de novo* formation of nuclear and cytoplasmic domains can be triggered by increased levels of the respective RNA and proteins. In HeLa cells, the overexpression of wild-type SMN results in an increased number of nuclear bodies and triggers the accumulation of this protein in distinct cytoplasmic clusters (Shpargel *et al.*, 2003; Sleeman *et al.*, 2003). A similar phenomenon has been reported in differentiating rat neurons, in which the transient expression of green fluorescent protein–SMN causes the formation of numerous SMN-rich cytoplasmic foci, which do not further interfere with cell differentiation (Navascues *et al.*, 2004).

It was demonstrated here that the formation of CsBs depends on the ratio between *de novo* U snRNA and Sm protein synthesis and the steady-state snRNP pool in the nucleus. snRNPs are distinctly stable macromolecules, and defects in the proper regulation of their assembly and rate of formation can cause major disorders in cell functioning (Strzelecka *et al.*, 2010*b*; Shukla and Parker, 2014). This stability is the main reason why it is essential for the cell to maintain the proper level of snRNAs relative to the protein components of the snRNP. It is proposed here that CsBs function as sites of kinetic coordination of the *de novo* synthesis of splicing elements and could comprise sites of specific control points to ensure the proper assembly of the Sm core on U snRNA during intensive synthesis.

The quantitative analysis indicated that, during the fourth cycle, the level of newly synthesized snRNPs is the highest compared with the nuclear steady-state pool (Fig. 5), which triggers snRNP-rich cytoplasmic body formation. These data indicate that CsBs formed during this cycle serve as self-organizing spatial platforms of assembly, which would facilitate the effectiveness of the process. This finding is similar to the recent model of *de novo* formation of nuclear domains involved in RNP processing. It has been postulated that CBs assemble by self-organization and form as local concentrations of macromolecules, such as coilin and Sm proteins (Misteli, 2007; Kaiser *et al.*, 2008). Furthermore, it has been demonstrated that *de novo* formation of CBs can be induced by increased nuclear snRNP levels (Lemm *et al.*, 2006; Strzelecka *et al.*, 2010*a*) and enhanced Sm protein expression (Sleeman *et al.*, 2001). Nevertheless, in cells with low metabolic activity, the processes of snRNP assembly and maturation can occur without the presence of CBs (Deryusheva and Gall, 2009; Deryusheva *et al.*, 2012). Thus, the occurrence of cytoplasmic bodies during periods of increased splicing element expression reflects the role of these dynamic structures in the enhancement of the effectiveness of snRNP assembly, which, in periods of moderate *de novo* synthesis (below a certain threshold), occurs diffusely within the cytoplasm.

### Periodic expression of splicing elements during diplotene

The occurrence of five cycles of snRNP synthesis during diplotene in larch microsporocytes correlates with the occurrence of five cycles of increased general transcriptional activity of these cells (Kołowerzo-Lubnau *et al.*, 2015). Thus, it is most likely that an increased level of investigated U snRNPs is related to the maturation and storage of splicing elements for nascent pre-mRNA processing during five transcriptional rounds. This is the case for the first half of diplotene (cycles 1–3), when an increased level of polyadenylated RNA that colocalizes with newly formed transcripts was identified. Simultaneously, increased levels of the hyperphosphorylated form of RNA polymerase II were noted, which is an indicator of nascent pre-mRNA elongation (Kołowerzo-Lubnau *et al.*, 2015). Moreover, during this diplotene stage, microsporocytes exhibit an increased level of ribosomal RNA synthesis (Smoliński *et al.*, 2007; Smoliński and Kołowerzo, 2012), which correlates with increased *de novo* protein synthesis within the cytoplasm (Kołowerzo-Lubnau *et al.*, 2015). Thus, abundant snRNP synthesis during the first half of diplotene results from the requirement of transcriptionally active cells for rapid and efficient pre-mRNA processing. Strzelecka *et al.* (2010*b*) identified a relationship between the levels of functional snRNP synthesis and the survival of metabolically active embryos, which was linked with effective pre-mRNA splicing.

During the late diplotene stage, two significant increases in U4 snRNA, U5 snRNA, and Sm protein levels were identified. Increased amounts of m3G-capped snRNAs and U2 snRNA are simultaneously localized (Kołowerzo-Lubnau *et al.*, 2015). Similar to previous stages, the general transcriptional activity is maintained at a high level in microsporocytes; however, the level of polyadenylated RNA decreases considerably (Kołowerzo-Lubnau *et al.*, 2015). The significant drop in the colocalization of polyadenylated RNA with newly formed transcripts, as well as the low level of RNA polymerase II, indicate that during the second half of diplotene, mRNA synthesis is reduced. Thus, increased levels of general transcription refer to other RNAs. This finding is confirmed by quantitative measurements of both U snRNA (Fig. 1) and rRNA levels (Smoliński *et al.*, 2007; Kołowerzo-Lubnau *et al.*, 2015), which indicate significant increases in these transcripts during the second half of diplotene. Thus, the *de novo* synthesis of snRNP in this period is not caused by increased pre-mRNA expression, which was the case for the early stages of diplotene, when the cell doubles its volume (Kołowerzo-Lubnau *et al.*, 2015). Elevated levels of U snRNPs probably serve as a storage pool of the splicing machinery, which would be passed to daughter cells after meiotic division. These daughter cells, which are referred to as microspores, have been implied to exhibit high transcriptional activity (Mascarenhas, 1975; Tupy *et al.*, 1983; Bednarska, 1984). Additionally, they accumulate abundant amounts of snRNA in the nucleus during the early stages of development (Zienkiewicz *et al.*, 2008). The phenomenon of passing U snRNPs to daughter cells after division has been demonstrated previously in both somatic (Ferreira *et al.*, 1994; Sleeman and Lamond, 1999) and transcriptionally silenced early embryonic cells (Ferreira and Carmo-Fonseca, 1995; Ferreira and Carmo-Fonseca, 1996; Strzelecka *et al*, 2010*b*). Sleeman *et al.* (2003) demonstrated that, during mitosis in HeLa cells, CBs that contain both Sm proteins and m3G-capped U snRNA are passed to daughter nuclei after division. Additionally, larch tetrads exhibit an increased concentration of snRNPs prior to the initiation of transcriptional activity. Furthermore, in mature larch microspores, the level of these molecules is significant (Niedojadło and Górska-Brylass, 2003).

### Insights into the cytoplasmic pools of Sm proteins

The present studies indicate that U snRNP accumulation within distinct cytoplasmic bodies occurs exclusively during the fourth cycle of synthesis (i.e. both U4 snRNA and Sm proteins were enriched in CsBs only during this cycle). During the first, second, and fifth cycles, noticeable cytoplasmic clusters were identified enriched in Sm proteins, lacking U4 snRNA. It cannot be excluded that these accumulations contain other types of spliceosomal U snRNAs. However, this possibility is unlikely, considering the fact that both U4 and U5 exhibited comparable localization patterns during the investigated diplotene stage and did not localize to CsBs during the same stages. These data imply that this pattern is similar for all U snRNA types.

The observed cytoplasmic accumulations of Sm could comprise the sites of synthesis and/or storage of these proteins. Prior to binding to snRNA, Sm proteins form primary RNA-free oligomers, including E–G–F, D1–D2, and B/B’–D3 (Hermann *et al.*, 1995; Raker *et al.*, 1996; Kambach *et al.*, 1999; Will and Lührmann, 2011*b*; Matera and Wang, 2014). Cytoplasmic accumulations of Sm proteins in larch microsporocytes may thus comprise putative sites of Sm oligomer storage, which are subsequently released to the cytoplasm and recruited on nascent U snRNA. In addition, they could serve as sites of Sm storage in the form of conjugates with PICln and/or methylosome in periods of increased protein expression, which would prevent uncontrolled binding to RNAs (Friesen *et al.*, 2001; Meister *et al.*, 2001; Pellizzoni *et al.*, 2002; Yong *et al.*, 2004). Alternatively, they could serve as sites of Sm activation and further interaction with SMN. Previous studies have demonstrated a relationship between SMN expression and the integrity of U bodies involved in cytoplasmic snRNP assembly/storage in *Drosophila* (Lee *et al.*, 2009). Sleeman *et al.* (2003) demonstrated that increased fluorescent protein–SMN expression leads to cytoplasmic accumulation of SMN-complex proteins. Furthermore, these accumulations also contain SIP1/Gemin2 and Sm proteins; however, they do not contain m3G-capped RNA. Further analysis regarding the localization of factors such as PICln, methylosome, and SMN would enable the verification of the role of Sm-rich cytoplasmic clusters. Nevertheless, no plant homologue of SMN protein has been described to date.

It cannot be ruled out that part of the distinct cytoplasmic Sm-rich foci identified are not involved in snRNP synthesis. Several studies regarding the participation of canonical Sm proteins in cytoplasmic processing and transport of mRNA have been described previously in animal germ lines and early embryonic cells (Bilinski *et al.*, 2004; Gonsalvez *et al.*, 2010).The latest research from Lu *et al.* (2014) has demonstrated that within cells, Sm proteins are associated with three major types of RNA: (i) snRNA, (ii) scaRNA, and (iii) mRNA. Seventy-two types of mRNAs associated with Sm for *Drosophila* and 30 types for HeLa cells have been demonstrated to be related to mitochondria, ribosomes, and translation. The interaction between Sm proteins and mRNA in a splicing-independent manner in these models probably occurs within specialized cytoplasmic domains, referred to as germ granules (Barbee *et al.*, 2002; Chuma *et al.*, 2003; Bilinski *et al.*, 2004). In *Caenorhabditis elegans*, P granules contain Sm proteins but not the other splicing elements, which indicates a new role of these proteins not related to splicing (Barbee *et al.*, 2002). Studies have confirmed that the suppression of Sm expression results in P granule dispersion (Barbee *et al.*, 2002). One cannot exclude the possibility that in larch microsporocytes, which are male germline cells, Sm protein accumulation within cytoplasmic clusters is involved in mRNA processing. This finding is supported by the analysis of colocalization of Sm proteins with U4 snRNA, which indicated that a considerable portion of cytoplasmic Sm does not colocalize with snRNA transcripts (70–99% depending on the cycle; Fig. 5). Moreover, our preliminary data indicated the cyclic occurrence of cytoplasmic clusters of Sm proteins that colocalize with polyadenylated RNA (M. Hyjek, unpublished observations). Further investigation of these Sm-rich cytoplasmic bodies lacking snRNA will enable an explanation of their function.

Assuming that Sm proteins concentrated in cytoplasmic bodies formed in the first, second, and fifth cycles function in processes not related to snRNP maturation, one must expect that during these periods, two distinct pools of canonical Sm proteins are present in the cytoplasm. The fraction accumulating within cytoplasmic clusters presumably plays a role in snRNP-independent processes, whereas the fraction dispersed diffusely is involved in snRNP assembly. This proposed model is supported by quantitative analysis. During each cycle, there is a strong correlation between the decrease in the cytoplasmic levels of Sm proteins and U4 snRNA (Fig. 1, asterisks, *r*=0.81). Correspondingly, a significant increase in both investigated splicing elements was noted in the nucleus, which was also strongly correlated (*r*=0.88). These data confirm that Sm-associated U snRNAs are reimported to the nucleus. Furthermore, the colocalization analysis of both molecules in the cytoplasm demonstrated that the percentage of U4 snRNA that colocalizes with Sm proteins is always significantly increased compared with the percentage of Sm proteins that colocalize with U4 snRNA (40–80% higher, depending on the cycle; Fig. 5). Thus, in the cytoplasm, there is a pool of Sm proteins not correlated with snRNAs. Based on these results, it is proposed here that there are several cytoplasmic pools of canonical Sm proteins in the cell, which are involved in independent processes of the regulation of distinct RNA types.

### Conclusions

In conclusion, it has been demonstrated for the first time, to the best of our knowledge, that under physiological conditions, the same cell model may establish two distinct spatial manners of cytoplasmic snRNP assembly: it can occur diffusely within the cytoplasm or in distinct, spatially separated microdomains. This manner is dependent on the rate of *de novo* formation of snRNP in relation to the steady state of these particles within the nucleus. Many studies have indicated that the pattern of snRNP cellular distribution is complex and highly ordered. It has been demonstrated that this localization reflects the hierarchical pathway of snRNP guidance and assembly, which is essential for the proper functioning of splicing machinery. Our studies have demonstrated that, similar to the nuclear steps of maturation, cytoplasmic snRNP assembly and processing may also be strictly spatially regulated and involve specialized distinct non-membranous structures. This type of highly ordered trafficking pathway of snRNPs in both cellular compartments (nucleus and cytoplasm) comprises an interesting model for studies regarding the dynamics and mechanisms that drive and coordinate RNP expression within a cell.

## Supplementary data

Supplementary data are available at *JXB* online.

Supplementary Fig. S1. Double labelling of U5 snRNA and Sm proteins during the fourth cycle of synthesis.

Supplementary Fig. S2. Double labelling of U5 snRNA and Sm proteins during the fifth cycle of synthesis.

Supplementary Fig. S3. Analysis of the fluorescence intensity of U4 snRNA and Sm proteins in the cytoplasm of larch microsporocytes during the stages of cytoplasmic snRNP assembly.

Supplementary Data
