# Supplementary material for: Spatial regulation of cytoplasmic snRNP assembly at the cellular level
Source: J Exp Bot. 2015 Aug 27;66(22):7019–30. doi: 10.1093/jxb/erv399 (PMC4765780; doi:10.1093/jxb/erv399)
Supplement: Supplementary Data [file supp_66_22_7019__index.html]

Spatial regulation of cytoplasmic snRNP assembly at the cellular level — Spatial regulation of cytoplasmic snRNP assembly at the cellular level — Supplementary Data 

# Spatial regulation of cytoplasmic snRNP assembly at the cellular level

## Supplementary Data

Data files

- Supplementary Data - Supplementary Data
