# Supplementary material for: Spatial regulation of cytoplasmic snRNP assembly at the cellular level
Source: J Exp Bot. 2015 Aug 27;66(22):7019–30. doi: 10.1093/jxb/erv399 (PMC4765780; doi:10.1093/jxb/erv399)
Supplement: Supplementary Data [file supp_erv399_jexbot150888_file001.pdf]

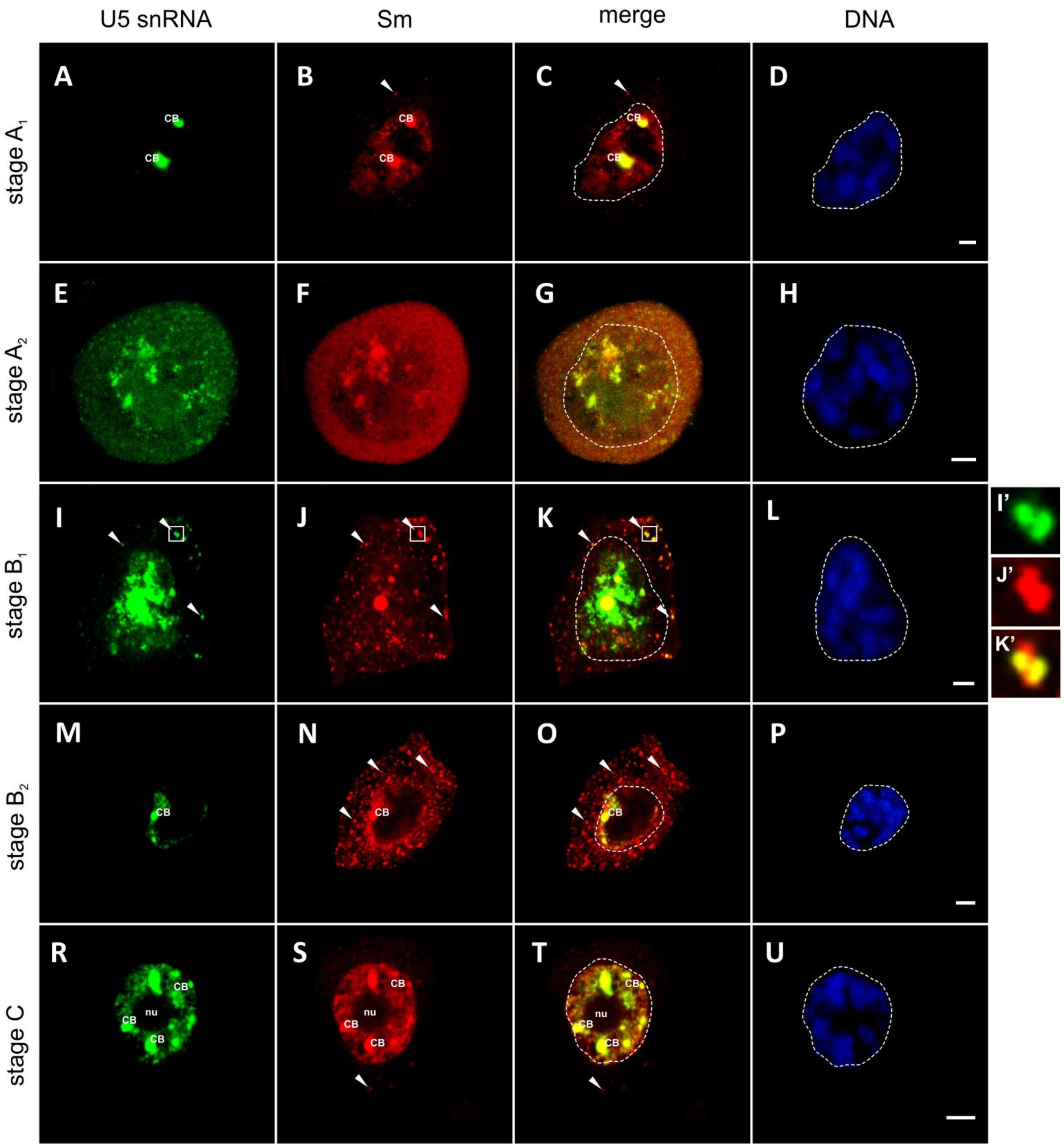

Figure S1. Double labeling of U5 snRNA and Sm proteins during the fourth cycle of synthesis.

Stages A and B are divided in two sub-stages referred to as A_1 and A_2 for stageA and B_1 and B_2 for stage B because of the distinct snRNP patterns of localization.

A-D Stage A_1. Nuclear U5 snRNA fluorescence is visible mostly within Cajal bodies (**A**), which colocalizes with the Sm signal (**B, C**). Additionally, distinct foci of Sm signal accumulation are observed in the cytoplasm (**B**, arrowhead), devoid of U5 snRNA signal (**C**, arrowhead). **E-H** Stage A_2. The U5 snRNA signal localizes to the entire nucleus, with noticeable irregular accumulations of transcripts (**E**) devoid of the Sm signal (**F**, **G**). **I-K** Stage B_1. The cytoplasmic pool of U5 snRNA is localized in large cytoplasmic clusters (**I**, arrowheads, **I'**), which colocalize with Sm proteins (**J, K**, arrowheads, **J'**, **K'**). **M-P** Stage B_2. nuclear U5 snRNA and Sm staining shows distinct accumulation of these molecules in individual Cajal body that forms in close proximity to the nuclear envelope (**M, N, O**). No cytoplasmic clusters of U5 snRNA fluorescence are visible (**M**), whereas Sm staining still exhibits numerous Sm-rich cytoplasmic granules (**N**, **O**, arrowheads). **R-U** Stage C. The nuclear signal of U5 snRNA and Sm fluorescence shows a dispersed pattern of localization within the nucleoplasm (**R**, **S**); the Cajal bodies are located throughout the whole nucleus and are frequently associated with the nucleolus (**R, S, T**). In the cytoplasm, there are Sm-containing clusters that remain visible (**S, T**, arrowheads), lacking U5 snRNA (**R**). The corresponding DAPI images were collected using wide field fluorescence (**D, H, L, P, U**). CB – Cajal body, nu - nucleolus. Bars 10 µm.

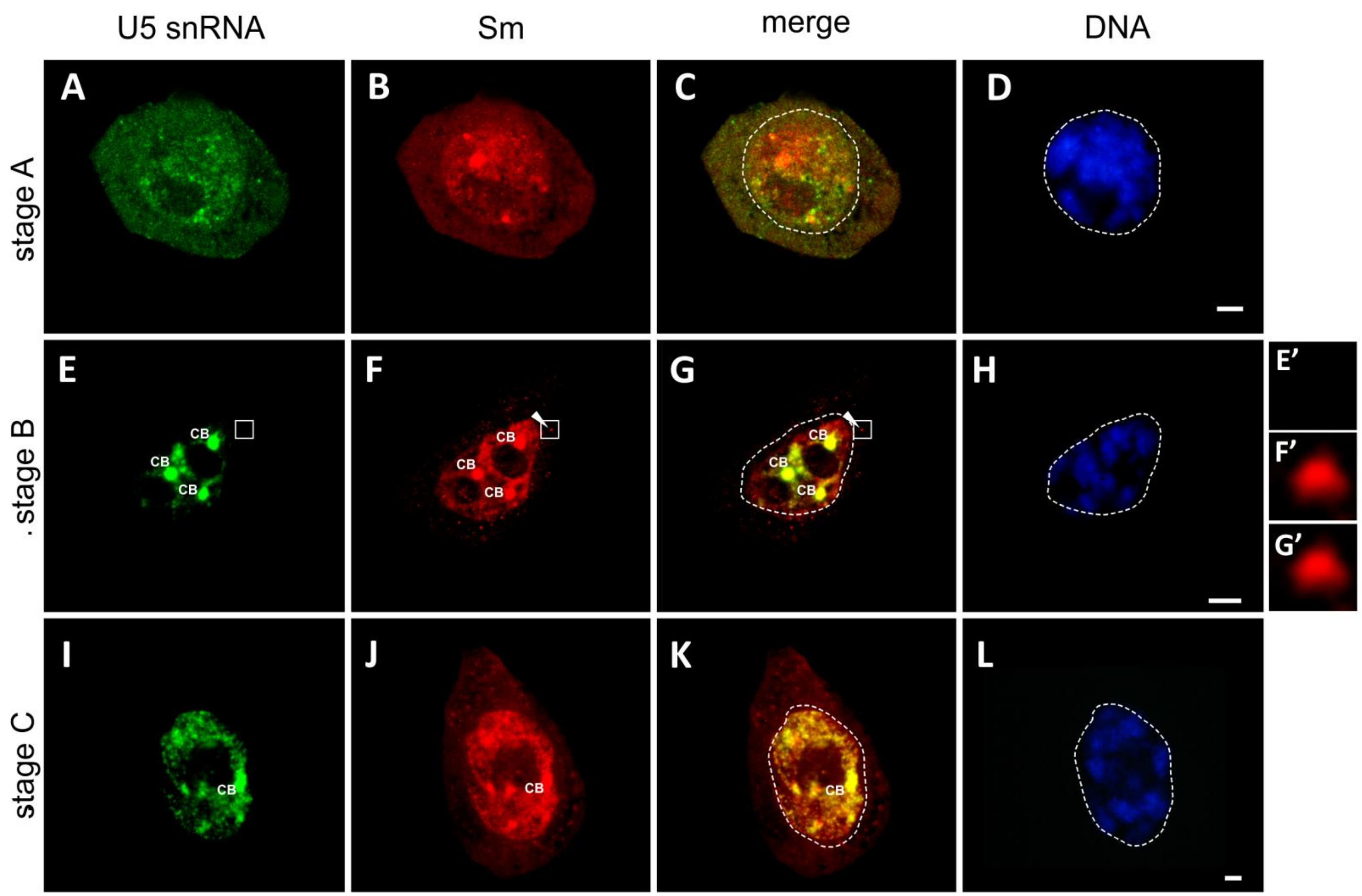

Figure S2. Double labeling of U5 snRNA and Sm proteins during the fifth cycle of synthesis.

A-D Stage A. Nuclear U5 snRNA staining is visible (A), with a noticeable portion of nucleoplasmic signals devoid of Sm accumulation (B, C). The cytoplasmic signal from Sm localization is dispersed throughout the cytoplasm, and no accumulation of Sm in distinct clusters is present (B). E-H Stage B. The U5 snRNA signal shows diffused pattern of localization within the nucleoplasm (E); it is also enriched in numerous Cajal bodies, which colocalizewith Sm staining (E, F, G). The cytoplasmic Sm localization is dispersed with discrete accumulations (F, G, arrowheads, F', G'), lacking U5 snRNA (E, E'). I-L Stage C. An increased level of nuclear Sm staining is visible, which colocalizes with U5 snRNA in both the nucleoplasm and Cajal body (I, J, K). The corresponding DAPI images were collected using wide field fluorescence (D, H, L). CB – Cajal body. Bars 10 μm.

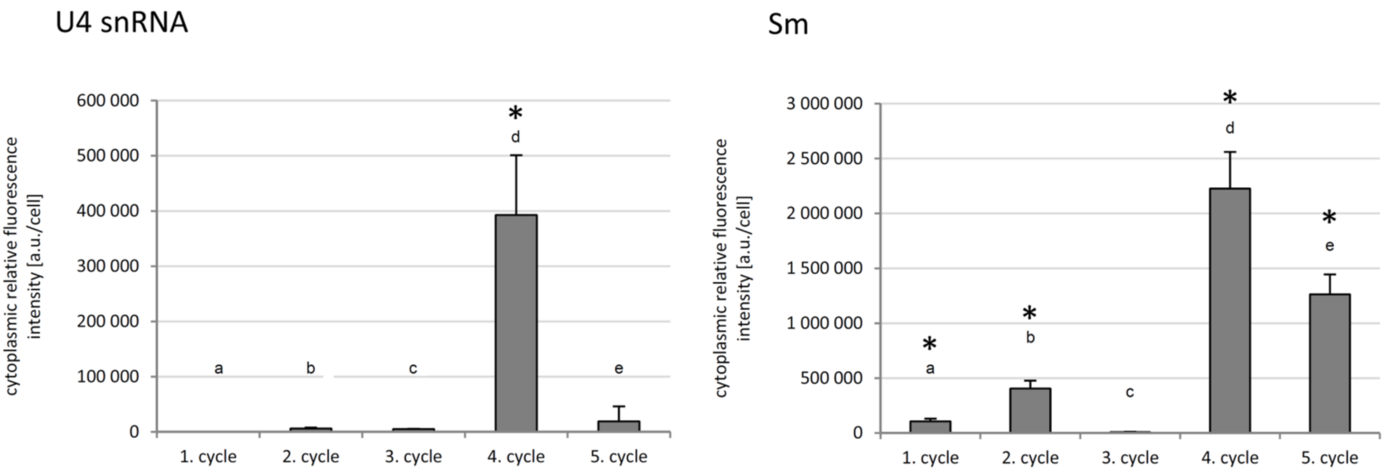

Figure S3. Analysis of the fluorescence intensity of U4 snRNA and Sm proteins in the cytoplasm of larch microsporocytes during the stages of cytoplasmic snRNP assembly.

Asterisks represent the stages of accumulations of investigated molecules in distinct cytoplasmic clusters. For the U4 snRNA, the significant differences between the signal intensities during specific stages are as follows: $p<0.05$ d-e, $p<0.001$ a-b, a-c, a-d, a-e, b-d, c-d. For the Sm proteins, significant differences between the signal intensities during specific stages are as follows: $p<0.01$ a-b, $p<0.001$ a-c, a-d, a-e, b-c, b-d, b-e, c-d, c-e. Error bars represent the s.e.m.
